# The Role of Diacylglycerol Kinase in the Amelioration of Diabetic Nephropathy

**DOI:** 10.3390/molecules27206784

**Published:** 2022-10-11

**Authors:** Daiki Hayashi, Yasuhito Shirai

**Affiliations:** Department of Applied Chemistry in Bioscience, Graduate School of Agricultural Science, Faculty of Agriculture, Kobe University, Kobe 657-8501, Japan

**Keywords:** diabetic nephropathy, protein kinase C, diacylglycerol kinase, 67 kDa laminin receptor

## Abstract

The drastic increase in the number of patients with diabetes and its complications is a global issue. Diabetic nephropathy, the leading cause of chronic kidney disease, significantly affects patients’ quality of life and medical expenses. Furthermore, there are limited drugs for treating diabetic nephropathy patients. Impaired lipid signaling, especially abnormal protein kinase C (PKC) activation by de novo-synthesized diacylglycerol (DG) under high blood glucose, is one of the causes of diabetic nephropathy. DG kinase (DGK) is an enzyme that phosphorylates DG and generates phosphatidic acid, i.e., DGK can inhibit PKC activation under diabetic conditions. Indeed, it has been proven that DGK activation ameliorates diabetic nephropathy. In this review, we summarize the involvement of PKC and DGK in diabetic nephropathy as therapeutic targets, and its mechanisms, by referring to our recent study.

## 1. Diabetes and Diabetic Nephropathy

Diabetes is a chronic metabolic disease that causes high blood glucose levels, resulting in serious health problems and death. In 2021, the number of people living with diabetes globally was estimated to be 536.6 million (prevalence: 10.5%), and this number is expected to reach 783.2 million (prevalence: 12.2%) by 2045 [1]. Diabetes significantly increases the risk of various health issues such as heart attacks, strokes, and infections compared to healthy individuals [2,3]. Diabetes also causes serious vascular complications. Diabetic retinopathy, neuropathy, and nephropathy are major microvascular complications of diabetes caused by microangiopathy. These complications lead to blindness, gangrene, and end-stage renal disease, respectively. Diabetic nephropathy (DN) is the leading cause of chronic kidney disease (CKD) in the world, leading to the need for dialysis, reducing patients’ quality of life, and increasing medical care expenses.

DN causes glomerular filtration failure that leads to uremia in one in three diabetic patients after several years of latency [4]. Since a high blood glucose level is the fundamental cause of the complications, delaying the onset of DN by controlling the blood glucose level is the primary treatment strategy [5]. Recently, many drugs to control blood glucose levels, such as insulin, sodium-glucose co-transporter-2 (SGLT-2) inhibitors, and glucagon-like peptide-1 (GLP-1) receptor agonists, have become available. It is also well recognized that the renin-angiotensin-aldosterone system (RAAS), which controls arterial pressure, plays a pivotal role in the pathogenesis of DN. So far, drugs targeting the RAAS, such as angiotensin-converting enzyme (ACE) inhibitors and angiotensin II receptor blockers (ARBs), have been the first choice for preventing and delaying the onset of DN. However, there are limits to the drugs available for DN, and there are no specific therapeutics for ameliorating DN once hyperglycemia and high blood pressure become uncontrolled. Although controlling blood glucose levels is the most simple and effective approach to delaying the onset of microvascular complications, and inhibiting RAAS is effective for DN, finding a novel target with a renoprotective effect was necessary. Therefore, to develop a novel drug for DN, it was vital to understand the mechanisms of the development and progression of DN and find an effective therapeutic target.

## 2. Hyperglycemia Impairs Lipid Signaling

**Figure 1:**
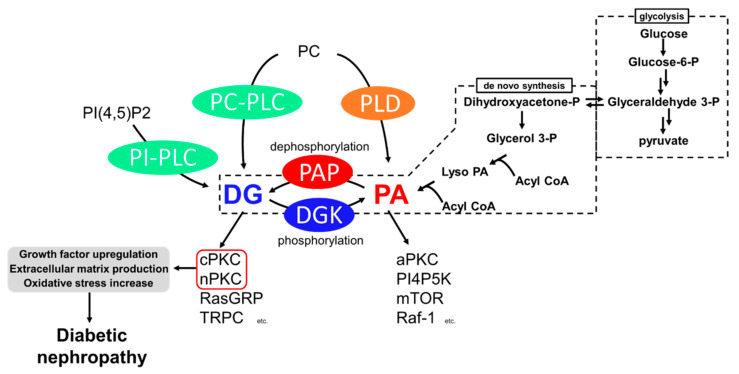
**Generation pathways of DG and pathways leading to diabetic nephropathy.** P: phosphate.

Among the causes of DN, impaired lipid signaling, especially diacylglycerol (DG) signaling, is well known. Generally, DG is produced from phosphatidylinositol 4,5-bisphosphate [PI(4,5)P2] and phosphatidylcholine (PC) by the action of phosphoinositide-specific phospholipase C (PI-PLC) and PC-specific PLC (PC-PLC) upon various growth factors and T-cell receptors (TCR) (Figure 1) [6,7,8]. In addition, DG is generated from the dephosphorylation of phosphatidic acid (PA) by phosphatidate phosphohydrolases (PAPs), including lipins [9,10]. PA is generated by hydrolyzing PC by phospholipase D (PLD) [11] and de novo synthesis from glycerol [12]. Glyceraldehyde 3-phosphate, an intermediate of glycolysis, is reversibly isomerized into dihydroxyacetone-phosphate and produces glycerol 3-phosphate. The glycerol 3-phosphate undergoes acylation and generates phosphatidic acid (PA), and dephosphorylates PA into DG (Figure 1). Under hyperglycemic conditions, excess blood glucose is uptaken intracellularly by the glucose transporter (GLUT) [13,14], and excess intracellular glucose results in DG production by de novo synthesis [12]. Both DG and PA are important intermediates for membrane lipids and are bioactive lipids involved in various signal transductions. For example, DG regulates conventional and novel protein kinase C (cPKC and nPKC) [15,16], Ras guanyl nucleotide-releasing protein (RasGRP) [17], and transient receptor potential channel (TRPC) [18]. PA regulates atypical PKC (aPKC) [19], phosphatidylinositol-4-phosphate 5-kinase (PI4P5K) [20], Raf-1 kinase [21], and mammalian target of rapamycin (mTOR) [22]. Therefore, abnormal DG production causes severe changes in their signaling pathways. The abnormal activation of cPKC and nPKC has been reported as a cause of DN.

## 3. Diabetic Nephropathy and PKC

PKC is a family of serine/threonine kinases, and nine mammalian isozymes are categorized into three groups based on their domain structure and characteristics [23]. cPKC (PKCα, β, γ) possesses diacylglycerol (DG) binding C1 domains (C1A and C1B) and a Ca^2+^ binding C2 domain, and their activity depends on DG and Ca^2+^ [24,25]. nPKC (PKCδ, ε, η, θ) has a slightly different domain composition than cPKC and lost Ca^2+^ dependency [26]. The DG-responsive characteristics of cPKC and nPKC are crucial to the pathogenesis of DN. The final subfamily requires neither Ca^2+^ nor DG for its activity and was cloned and classified as aPKC (PKCι, ζ). It is activated by PA [27,28]. These PKCs phosphorylate many proteins, including receptors, transporters, kinases, and phosphatases; thus, PKCs play a central role in cell signaling [23]. To date, the increase in DG mass and the abnormal activation of cPKC and nPKC under hyperglycemic conditions have been observed in vivo and in vitro [29,30,31], and evidence supports the idea that the abnormal activation of cPKC and nPKC is one of the causes of the development and progression of diabetic nephropathy [32,33,34,35].

Abnormally activated PKC causes various biological changes, such as growth factor upregulation, extracellular matrix production, and oxidative stress increase (Figure 1). Among PKC isozymes, activation of PKCα, β (cPKC) and PKCε, δ (nPKC) have been observed in DN [28,34,35,36,37,38]. One of the mechanisms underlying how PKC activation causes DN is related to various growth factors [39]. The upregulation of transforming growth factor-β (TGF-β) signaling, a profibrotic cytokine, is considered a critical pathogenic and progression factor of DN by activating various signal transductions, expression of the extracellular matrix, and reactive oxygen species production [40,41]. It has been reported that diabetes-induced activation of PKCβ and PKCδ upregulates TGF-β expression and the sensitivity of the cell against TGF-β, promoting the production of the extracellular matrix [42,43]. In addition to TGF-β, connective tissue growth factor (CTGF) and vascular endothelial growth factor (VEGF) are also recognized as key factors in DN progression [37]. CTGF is known to promote extracellular matrix production, leading to fibrosis [44]. VEGF is known as an angiogenic factor promoting vascular endothelial cell proliferation and pathological angiogenesis [45]. Several studies have reported that activation of PKCα, β, and ε upregulates VEGF expression [46,47], and PKCβ upregulates CTGF expression [34]. Therefore, PKC inhibition can normalize these growth factor expressions and signaling and ameliorate DN. Moreover, PKCα and β are associated with reactive oxygen species (ROS) production by activation of NADPH oxidase, causing renal injury [34,48].

To develop drugs targeting abnormal PKC activation, a PKCβ-specific inhibitor, ruboxistaurin, was developed and showed a renoprotective effect in several animal studies [49,50]. However, in human clinical trials, although ruboxistaurin showed some renoprotective effect in diabetes patients, statistical significance was not observed compared to placebo patients [35]. Furthermore, since DG–PKC pathway activation is a common event in other diabetic microvascular complications, ruboxistaurin underwent phase 3 clinical trials for diabetic retinopathy. Nevertheless, the trial failed due to the insignificance of its effect on retinopathy compared to the placebo group [51]. Taken together, the upregulation of the DG–PKC pathway in diabetes is one of the key events in the pathogenesis and progression of DN. However, since several independent PKC isoforms are involved in complex and multifactorial pathways, developing a drug targeting PKC is not simple. For example, PKCα deficiency prevents albuminuria by reducing VEGF. However, glomerular hypertrophy is not inhibited due to no change in TGF-β expression [33]. In contrast, PKCβ deficiency prevents glomerular hypertrophy by reducing TGF-β but not albuminuria in mice [34,52]. Therefore, inhibiting a single PKC might be insufficient to prevent DN. One can overcome this by developing a pan-inhibitor for cPKC and nPKC, or a combination of several specific inhibitors, though issues with side effects may emerge.

## 4. Diabetic Nephropathy and DGK

DG kinase (DGK) is a family of enzymes that converts DG into PA by phosphorylation [53]. As illustrated in Figure 1, DGK is a central regulator in various signaling pathways related to DG and PA [54]. Since DGKs consume DG, DGK is recognized as an indirect inhibitor of cPKC and nPKC. So far, 10 subtypes of mammalian DGKs have been discovered and divided into Type I~V based on their structural characteristics [54,55,56,57]. All mammalian DGKs have several C1 domains homologous to the one of PKCs at the nitrogen ends of catalytic domains (CD). Type I DGKs (DGKα, β, γ) have a recoverin homology (RVH) domain and two EF-hand motifs, which bind to Ca^2+^ at the nitrogen end of the two C1 domains [58]. Type II DGKs (DGKδ, η, κ) are characterized by a split CD, with a pleckstrin homology domain (PH domain) at the N terminus. Among Type II DGKs, DGKδ and η have a sterile α motif domain (SAM domain) at the C terminus [59,60,61]. DGKε has a simple structural feature composed of two C1 domains and a CD and is the only Type III DGK subtype [62]. Type IV DGKs (DGKζ, ι) possess ankyrin repeats at the C terminus, and residue between the C1 domains and the CD have homologs to the myristoylated alanine-rich C-kinase substrate phosphorylation site [63,64,65]. Type V DGKs (DGKθ) is the only subtype that has three C1 domains, and a PH domain overlapping the Ras-associating domain (RA domain) is localized between the C1 domains and the CD [66]. In addition, splicing variants to alter their domain composition has been reported in several subtypes.

Vitamin E is a hydrophobic antioxidant agent, and α-tocopherol is the most active vitamin E compound that humans preferentially absorb [67]. The main function of α-tocopherol is its antioxidant effect, preventing lipid peroxidation in the cell membrane [68]. In 1997, it was reported that α-tocopherol ameliorates the symptoms of DN by activating DGK and inhibiting glomerulus PKC in diabetic rats, suggesting the possibility of DGK as a therapeutic target for DN [69,70]. However, the subtype of DGK involved in the amelioration was unclear. In 2005, it was revealed that a Type I DGK, DGKα, is sensitive to α-tocopherol among the DGKs expressed in the murine glomeruli (DGKα, γ, δ, ε, and ζ) [71], i.e., in the cultured cell, α-tocopherol induced translocation of GFP tagged DGKα to the plasma membrane, which is the hallmark of DGK activation, while the other DGKs did not [72,73]. Although the primary function of α-tocopherol as a vitamin is its antioxidant effect, several studies have reported that the antioxidant effect of α-tocopherol is not related to PKC inhibition [74,75]. Indeed, it was revealed that the antioxidant effect of α-tocopherol is not necessary to activate DGKα [73]. There is a report showing that the antioxidant effect of α-tocopherol plays a protective role against the loss of activity of DGKα under the high glucose condition [76]. The detailed mechanisms of DGKα activation by α-tocopherol are discussed in a later section.

After discovering that α-tocopherol activates DGKα using DGKα deficient mice, it was proven that DGKα was involved in the renoprotective effect of α-tocopherol [77]. Interestingly, the study did not observe any renoprotective effect of α-tocopherol, including inhibition of albuminuria, in the DGKα deficient mice, indicating DGKα is responsible for the renoprotective effect of α-tocopherol. This suggests that the antioxidant effect of α-tocopherol itself was insufficient for the amelioration of DN, although reduction of oxidative stress is a key factor for the amelioration of DN [77,78]. Furthermore, in addition to DGKα, DGKδ has been implicated in the amelioration of DN by α-tocopherol [71]. The study showed that α-tocopherol increased the activity of both Ca^2+^ dependent (DGKα) and independent subtypes (DGKδ). However, whether DGKδ is involved in the amelioration of DN in vivo is still unknown. Further, DGKα and DGKδ are involved in insulin secretion and tolerance, respectively [79,80]. Therefore, activating DGKα and DGKδ might be beneficial to diabetes itself, in addition to DN.

Based on the many positive observations of the effect of α-tocopherol on DN in animal studies, a human trial was conducted. In the trial, patients with diabetes were supplemented daily with 400 IU of vitamin E, and outcomes of cardiovascular and DN were followed for 4.5 years. However, the supplementation of vitamin E did not have a positive effect on the outcomes, indicating that the renoprotective effect of α-tocopherol is not expressed in humans [81]. Since vitamin E is an essential micronutrient for humans and deficiency of vitamin E is rare in general, perhaps the additional effect of vitamin E was masked. Still, some studies have reported the renoprotective effect of α-tocopherol in humans, so the effect of tocopherols on DN needs more careful investigation [82,83]. In any case, it was still important to find other agents responsible for activating DGKα.

In addition to the effect of α-tocopherol, the involvement of DGKα in the renoprotective effect of green tea polyphenols has been unveiled. In the early 2000s, several studies reported the positive effect of green tea polyphenols on DN. Yokozawa et al. reported that supplementation of green tea polyphenols ameliorated albuminuria in diabetic rats [84]. Further, Yamabe et al. reported the positive effect of epigallocatechin gallate (EGCG), the most active green tea polyphenol, on the symptoms of diabetic nephropathy in rats, along with downregulation of TGF-β expression [85]. However, the underlying mechanisms of the renoprotective effect of the polyphenols have been elusive [86]. Recently, we discovered that EGCG activates DGKα, suggesting DGKα is involved in the renoprotective effect of green tea polyphenols [87]. Furthermore, using DGKα deficient mice, the effect of EGCG on DN was evaluated, and it was proven that DGKα is essential for the effect of EGCG on DN [88]. In the study, the amelioration effect of DN was assessed by oral administration of EGCG in DGKα deficient mice. These studies supported the idea that activation of DGKα is an attractive therapeutic target for DN.

Further, it was also found that the 3″-*O*-methylated EGCG (EGCG3″Me), which possesses high absorption in plasma, more effectively ameliorated DN than EGCG [88]. This indicated that green tea polyphenols, especially EGCG, could be a functional food for DN. Importantly, the significant DN ameliorating effect of EGCG was observed in a human trial [89]. It has been reported that EGCG can attenuate insulin resistance by inducing the translocation of GLUT4 [90,91]. Therefore, supplementation of EGCG may be recommended to prevent and ameliorate diabetes and DN. While there are two major issues that should be overcome for the use of EGCG for treating DN, daily consumption of green tea polyphenols can still be considered beneficial. One is toxicity, and the other is pharmacokinetics. High doses of EGCG are toxic [92], and the absorption of EGCG in plasma is low [93]. Although EGCG3″Me has improved pharmacokinetics, EGCG3″Me is a minor component of green tea polyphenols, and purification of EGCG3″Me is challenging. Therefore, finding an agent to overcome these issues is still required.

## 5. The Mechanisms of the Amelioration of DN by DGKα Activation

One of the mechanisms by which DGKα activation ameliorates DN is through PKC inhibition. While an inhibitor acts on a specific PKC subtype, DGKα activation can inhibit several PKCs simultaneously by reducing the amount of DG. This might be the benefit of targeting DGKα as a therapeutic target since several PKC species contribute to DN pathogenesis and progression. Indeed, it has been proven that α-tocopherol treatment inhibits the activation of both PKCβ and PKCα in diabetic mice kidneys [94]. It has not been determined whether DGKα can inhibit PKCε and PKCδ, but it is likely to inhibit both. Further, EGCG, a DGKα activating agent, ameliorates albuminuria with downregulation of TGF-β under diabetic conditions, indicating DGKα activation inhibits the downstream of PKCs, although direct evidence has not been obtained yet [95]. Based on this, DGKα activation can ameliorate DN by inhibiting various biological activities, followed by PKC activation.

**Figure 2:**
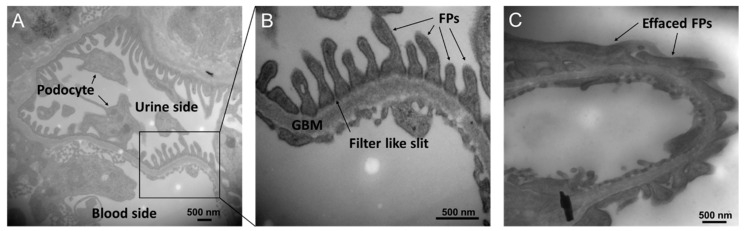
**Transmitted microscopy images of the slit membrane structure.** (**A**) A transmitted electron microscopy image of the mouse glomerulus. (**B**) A zoomed-in image of the slit membrane structure. (**C**) A collapsed slit membrane structure in streptozocin (STZ)-induced diabetic mice glomerulus.

Another important mechanism by which DGKα activation ameliorates DN is the protection of glomerular epithelial cells, and part of the effect might be independent of PKC inhibition. Glomeruli are composed of three types of cells: mesangial cells, vascular endothelial cells, and vascular epithelial cells (podocytes). It was found that DGKα is expressed abundantly in podocytes, suggesting DGKα contributes to the biological activity of podocytes [87]. The podocyte is a terminally differentiated cell that plays a critical role in filtration by forming a slit membrane structure that functions as a filtration barrier [96,97]. The slit membrane is a filter-like slit formed by interdigitated cell swellings called foot processes (FPs), lining the glomerular basement membrane (GBM) (Figure 2A,B). In many kidney diseases, including DN, barrier function is affected by FP flattening (called effacement) (Figure 2C) and loss of podocytes [98]. Our previous study revealed that DGKα activation protected against effacement and podocyte loss [77,88]. The inhibition of PKCα could be one of the mechanisms of its effect since it has been reported that PKCα promotes the endocytosis of nephrin, which localizes at tight junctions and plays a critical role in the barrier function of the slit membrane [99]. In addition to the tight junction, adhesion to GBM is also essential to maintaining the morphology of the slit membrane. α3β1 integrin is involved in adhesion, and it has been reported that DGKα can recruit β1 integrin to the peripheral membrane by generating PA [100,101,102,103]. Therefore, the recruitment of β1 integrin is suggested to be one of the mechanisms of the effect. Indeed, EGCG treatment prevents loss of focal adhesion in the high glucose cultured human podocyte [88]. Further, a human trial observed protection against podocyte apoptosis by green tea polyphenols in DN [89]. In cancer cells, it is well known that DGKα prevents apoptosis via various signaling pathways, followed by PA production [104,105,106], suggesting the involvement of DGKα in the prevention of apoptosis of podocytes by green tea polyphenols, especially EGCG. Taken together, DGKα activation ameliorates DN by not only inhibiting PKCs by reducing the amount of DG but also by generating PA activating downstream signaling pathways. In podocytes, DGKα activation plays a protective role in preventing loss and morphological changes to prevent DN.

## 6. The Mechanisms of DGKα Activation

**Figure 3:**
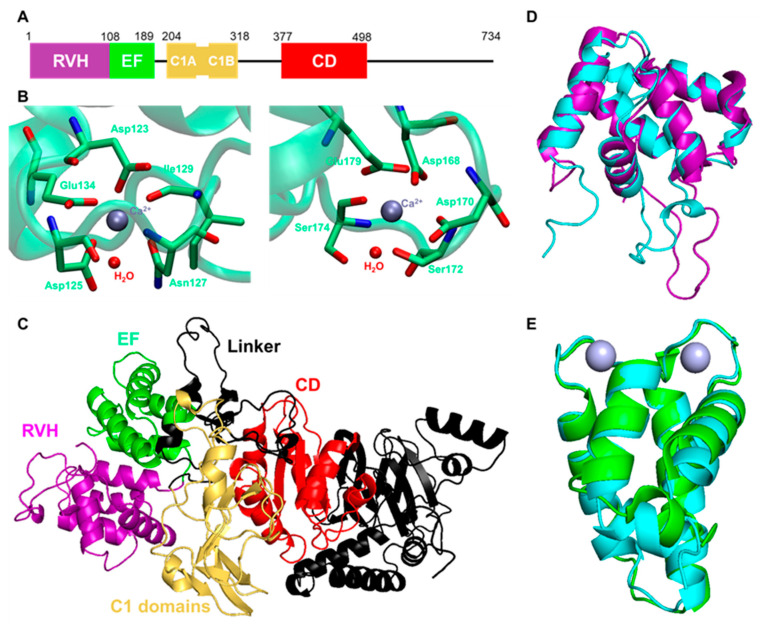
**The structural features of DGKα.** (**A**) Domain composition of human DGKα. (**B**) The residues contributing to the Ca^2+^ binding at EF-hand motifs of DGKα (PDB ID: 6IIE). (**C**) The predicted structure of human DGKα by AlphaFold2. The same color code as figure (**A**) was used. The comparisons of the predicted (**D**) RVH domain and (**E**) EF-hand motifs (cyan) with experimental structures (PDB ID: 1TUZ (purple) and 6IIE (green)). Gray particles are Ca^2+^.

Given that DGKα is a potential therapeutic target for DN, the activator of DGKα could be a good candidate for drugs for DN. As described before, DGKα possesses an RVH domain, two EF-hand motifs, and two C1 domains (Figure 3A). These domains have been suggested to regulate intracellular localization and activation of DGKα. Indeed, intramolecular interactions between EF-hand motifs and C1 domains are important to restrict the activation of DGKα. In the inactive state, EF-hand motifs intramolecularly interact with C1 domains, and Ca^2+^ activates DGKα by releasing EF-hand motifs from C1 domains [54,107,108]. Several lipids, such as phosphatidyl serine (PS) [108,109], phosphatidyl inositol phosphates (PIPs) [110], and sphingosines [111,112], are also known to enhance DGKα activity. Interestingly, the lipid-binding C1 domains are not responsible for PS-induced activation, and PS activates DGKα by interacting with its CD [109]. Activation mechanisms of PIPs and sphingosines have not been revealed yet. Still, these endogenous activators directly activate DGKα since they all show the activation effect in in vitro assays using purified DGKα. Developing a drug for DN based on a direct activator of DGKα is a simple and reasonable idea. So far, in vitro screening has been conducted to discover DGKα activators/inhibitors and has identified two activators [113,114]. However, there was still difficulty finding and evaluating direct activators via only in vitro screening since an active enzyme is used for the assay. The three-dimensional structure of DGKα can help to design the activator. However, the three-dimensional structure was not available, other than the nuclear magnetic resonance (NMR) structure of the RVH domain (PDB ID: 1TUZ). Recently, the crystal structure of the EF-hand motifs was solved by X-ray, with good resolution (PDB ID: 6IIE) [115]. As shown in Figure 3B, the crystal structure revealed that two Ca^2+^ ions bind to distinct binding sites, mainly composed of acidic amino acids. Since the Ca^2+^ binding of EF-hand motifs is critical for the activation of DGKα, it is now possible to design DGKα activators targeting EF-hand motifs based on the crystal structure.

Furthermore, recent rapid evolution in the prediction of the protein structure by deep learning provides the predicted structure of DGKα by AlphaFold2, as shown in Figure 3C [116,117,118]. Notably, although the prediction scores of linker regions between each domain are not so high as predicting the orientation of these domains, the structure of each domain seemed to be reliable enough. Indeed, the predicted RVH domain and EF-hand motifs overlapped well with the experimental structures, and the root-mean-square deviations (RMSD) were 1.229 and 1.076 Å, respectively (Figure 3D,E). In other words, taking advantage of the predicted structure makes it possible to understand the molecular basis of DGKα activation more deeply.

In addition to the direct activation, it is well known that tyrosine-phosphorylation regulates the localization and activation of DGKα [73,119,120]. Phosphorylation at Tyr335 on the linker region between the C1 domains and CD induces activation and translocation of DGKα to the plasma membrane and is regulated by Src family tyrosine kinase [73,119]. Our previous study revealed that α-tocopherol indirectly activates DGKα through phosphorylation at Tyr335 by Src family tyrosine kinases [73]. However, the specific receptor contributing to the α-tocopherol-induced activation of DGKα has not been determined, although the contribution of some receptors has been suggested. This is because no membrane receptor for α-tocopherol has been discovered, although its existence has been suggested [121].

Independently, it was discovered that EGCG indirectly activates DGKα, and the 67 kDa laminin receptor (67LR) mediates the activation [88]. 67LR is primarily recognized as a receptor for laminin, and various ligands such as Sindbis virus and pathogenic prion protein have been reported [122,123,124,125,126]. In 2004, it was reported that EGCG is one of the ligands for 67LR [127]. So far, it has been recognized that 67LR mediates many functions of EGCG, such as the anti-cancer effect and toll-like receptor 4 (TLR4) signaling inhibition [128,129,130]. However, there remains controversy over how 67LR activates the downstream signaling pathway, although several candidates have been suggested [128,131]. Our previous study indicated that Src family tyrosine kinase mediates the EGCG-induced DGKα activation, as with α-tocopherol, and interacts with 67LR [88]. Notably, another group also reported the implementation of Src family tyrosine kinase in EGCG-67LR signaling [132].

**Figure 4:**
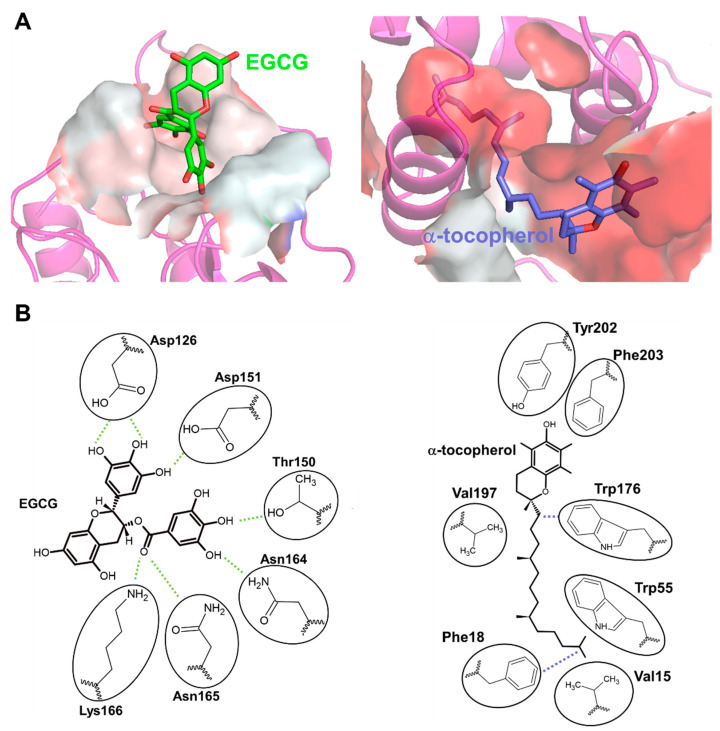
**Hydrophobicity of the EGCG and α-tocopherol binding site of 67LR.** (**A**) The amino acid residues of EGCG and the α-tocopherol binding site of 67LR are shown as surfaces colored by hydrophobicity (white: hydrophilic, red: hydrophobic. Green: EGCG, Blue: α-tocopherol). (**B**) The 2D binding mode of EGCG and α-tocopherol in each binding site. The 2D images are generated based on the binding mode of EGCG and α-tocopherol [133]. Green dashed lines indicate hydrogen bonds, and blue dashed lines indicate arene-hydrogen interactions with circled amino acids.

Based on the similarity between EGCG and α-tocopherol-induced DGKα activation, we considered whether 67LR also mediates α-tocopherol-induced DGKα activation and revealed that 67LR functions as an α-tocopherol receptor mediating DGKα activation [133]. Interestingly, both EGCG and α-tocopherol directly bind to 67LR, but they bind to different residues; the former was hydrophilic, while the latter was hydrophobic (Figure 4A) [133]. Notably, the detailed binding mode of EGCG and α-tocopherol to their binding pocket were revealed in the study by taking advantage of hydrogen-deuterium exchange mass spectrometry (HDX-MS) and molecular dynamics (MD) simulation [133]. According to the binding mode, the binding of EGCG to the site was ruled by hydrogen bonds through many hydroxy groups of EGCG (Figure 4B). α-tocopherol exerts its antioxidant effect through the hydroxyl moiety, but the binding of α-tocopherol was ruled by hydrophobic interactions, and the hydroxyl group was not part of the binding (Figure 4B). This suggests that the antioxidant ability of α-tocopherol was not related to the binding to 67LR. These studies clarified that both EGCG and α-tocopherol activate DGKα through 67LR. In other words, 67LR agonists can activate DGKα. Therefore, 67LR agonists could be another target for treating DN as an indirect activator of DGKα, although further studies are needed. Further, the study indicated that 67LR is the first discovered cell surface receptor for vitamin E and suggested that 67LR mediates some functions of vitamin E. Although further investigation on the physiological role of binding between 67LR and vitamin E is required, the agonist of 67LR can compensate for some functions of vitamin E.

## 7. Conclusions

**Figure 5:**
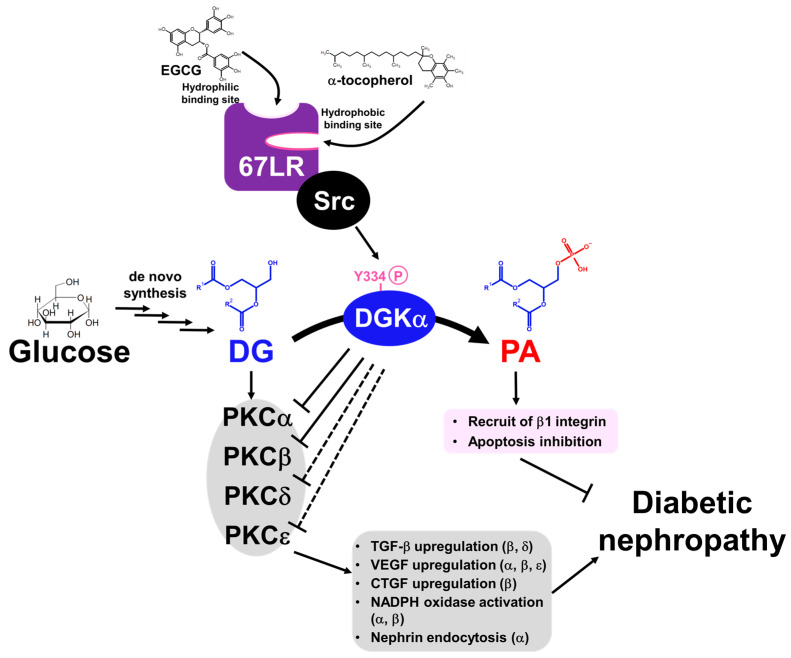
**Schematic figure of the progression and pathogenesis of DN by the DG-PKC pathway and activation mechanism of DGKα**.

Under hyperglycemic conditions, high blood glucose results in DG accumulation via de novo synthesis. The accumulation of DG abnormally activates PKCs, and the enhanced PKC pathways are one of the mechanisms for the development and progression of DN. PKC activation promotes biological changes leading to DN, such as growth factor expression followed by extracellular matrix production and oxidative stress (Figure 5). DGK is an enzyme that can inhibit PKC activity by converting DG into PA. So far, many studies have supported the idea that DGKα ameliorates DN by inhibiting abnormal activation of PKCs and by activating PA signaling (Figure 5). Notably, since DGKα activation can inhibit several PKC subtypes simultaneously, DGKα activation may be more effective than specific PKC inhibitors. So far, it has been proven that two food factors, α-tocopherol, and EGCG, ameliorated DN through DGKα activation in murine studies. Importantly, EGCG showed a positive effect on DN in humans, suggesting that EGCG is a functional food for DN by activating DGKα, although there are several issues, such as toxicity of EGCG for medical purposes. Therefore, the development of an effective DGKα activator is still needed.

Since DGKα inhibition can kill cancer cells and enhance T-cell function, the development of inhibitors has been focused on, and activators have received less attention [134,135,136]. Additionally, finding activators is generally challenging compared to finding inhibitors, which has delayed the development of activators. However, recently, state-of-the-art techniques have let us deeply understand the underlying mechanisms of DGKα activation and will enable the development of DGKα activators based on these mechanisms. Recently, it has been revealed that EGCG and α-tocopherol activate DGKα via 67LR binding, and their detailed binding mode has been elicited. Taken together, DGKα and its upstream 67LR are attractive therapeutic targets that normalize lipid signaling under diabetic conditions for DN, and 67LR agonists could be designed based on the binding mode of EGCG and α-tocopherol.

## Data Availability

Not applicable.
